# Internet of Things enabled open source assisted real-time blood glucose monitoring framework

**DOI:** 10.1038/s41598-024-56677-z

**Published:** 2024-03-14

**Authors:** Abubeker K. M, Ramani. R, Raja Krishnamoorthy, Sreenivasulu Gogula, Baskar. S, Sathish Muthu, Girinivasan Chellamuthu, Kamalraj Subramaniam

**Affiliations:** 1 Department of Electronics and Communication Engineering, Amal Jyothi College of Engineering (Autonomous), Koovappally, Kerala India; 2 Department of Computer Science and Engineering, P.S.R Engineering College, Sivakasi, Tamilnadu India; 3 Department of Electronics and Communication Engineering, Kalasalingam Academy of Research and Education, Krishnankoil, Tamilnadu India; 4 Department of Computer Science and Engineering, (Data Science), Vardhaman College of Engineering, Shamshabad, Hyderabad India; 5 Faculty of Engineering, Department of Electronics and Communication Engineering, Karpagam Academy of Higher Education, Coimbatore, Tamilnadu India; 6 Department of Orthopaedics, Government Medical College & Hospital, Karur, Tamilnadu India; 7 Department of Orthopaedics, Saveetha Medical College and Hospital, Saveetha Institute of Medical and Technical Sciences (SIMATS), Saveetha University, Chennai, Tamilnadu India; 8 Department of Biomedical Engineering, Karpagam Academy of Higher Education, Coimbatore, Tamilnadu India

**Keywords:** Biological techniques, Health care, Engineering

## Abstract

Regular monitoring of blood glucose levels is essential for the management of diabetes and the development of appropriate treatment protocols. The conventional blood glucose (BG) testing have an intrusive technique to prick the finger and it can be uncomfortable when it is a regular practice. Intrusive procedures, such as fingerstick testing has negatively influencing patient adherence. Diabetic patients now have an exceptional improvement in their quality of life with the development of cutting-edge sensors and healthcare technologies. intensive care unit (ICU) and pregnant women also have facing challenges including hyperglycemia and hypoglycemia. The worldwide diabetic rate has incited to develop a wearable and accurate non-invasive blood glucose monitoring system. This research developed an Internet of Things (IoT) - enabled wearable blood glucose monitoring (iGM) system to transform diabetes care and enhance the quality of life. The TTGOT-ESP32 IoT platform with a red and near-infrared (R-NIR) spectral range for blood glucose measurement has integrated into this wearable device. The primary objective of this gadget is to provide optimal comfort for the patients while delivering a smooth monitoring experience. The iGM gadget is 98.82 % accuracy when used after 10 hours of fasting and 98.04 % accuracy after 2 hours of breakfast. The primary objective points of the research were continuous monitoring, decreased risk of infection, and improved quality of life. This research contributes to the evolving field of IoT-based healthcare solutions by streaming real-time glucose values on AWS IoT Core to empower individuals with diabetes to manage their conditions effectively. The iGM Framework has a promising future with the potential to transform diabetes management and healthcare delivery.

## Introduction

Diabetes, a chronic metabolic disorder characterized by higher blood glucose levels, demands consistent monitoring and intervention. According to the World Health Organization (WHO), diabetes was the largest cause of mortality in 2021, accounting for 24.6 % of diabetic fatalities^1^. Maintaining appropriate Blood Glucose (BG) levels is of utmost importance in mitigating the potential risks associated with heart failure^2,3^, kidney diseases^4^, and retinal diseases^5^. Traditional glucose monitoring methods have often been cumbersome, inconvenient, and disconnected from patients' daily lives. Therefore, the timely detection of diabetes is of utmost importance to mitigate, monitor, or delay the progression of the condition and its related problems. Covid'19 is more likely to cause significant problems in people with diabetes^6,7^. Another disorder that affects blood glucose levels is hypoglycemia. Individuals diagnosed with diabetes experience a compromised capacity to manage blood glucose levels, leading to fluctuations characterized by hyperglycemia or hypoglycemia^8,9^. Non-invasive techniques obviate the necessity for uncomfortable finger punctures, rendering glucose monitoring more convenient and less obtrusive than traditional glucose monitoring. Utilizing a non-invasive approach presents a promising opportunity for continuously monitoring glucose levels, eliminating the necessity for regular blood collection. Using real-time data can offer an enhanced understanding of glucose variation, facilitating prompt modifications in medication, diet, and lifestyle.

According to recent research, individuals with diabetes mellitus (DM) are more likely to acquire pneumonia, and their death rate is significantly greater than that of non-diabetic patients^10,11^. Pre-existing diabetes has linked to variations in blood glucose levels in individuals with community-acquired pneumonia (CAP) and a high mortality rate^12^. A continuous glucose monitor (CGM) is an alternative for patients with hypoglycemia^13,14^. Non-invasive glucose monitors are in high demand since many patients are averse to repeatedly puncturing their fingertips to check their blood glucose levels. In addition, there are some issues with the precision of current CGM technology, so a non-invasive sensor that is more precise than current CGMs would be advantageous.

Recently, machine learning (ML), and artificial intelligence (AI) techniques has showcased the capabilities estimating blood glucose levels. These techniques utilize data obtained from non-invasive Wearable Devices (WD), which in turn enables the monitoring and management of individuals with diabetes using digital biomarkers^15,16^. The Vivovitals platform, developed by Sachmechi et al.^17^, serves as an additional tool to aid in the reduction of HbA1c levels in individuals diagnosed with uncontrolled type 2 diabetes mellitus. This platform is utilized in combination with lifestyle modifications and regular adjustments to antidiabetic drugs. Wearability and clinical accuracy are two critical components of this kind of device's design. Due to the near-field sensor's length, Kseniya Zavyalova et al.^18^ developed a device based on a flexible substrate. The sensor's extended near field and high electromagnetic field penetration depth were shown with a slot antenna combination. The technological advancement highlighted the potential of human biological fluids, including tears^19^, saliva^20^, and interstitial fluids^21^, as biomarkers for monitoring glucose levels. In a study by K.A. Saraswathi et al.^22^, a unique non-enzymatic electrochemical sweat sensor was developed for glucose monitoring using polyaniline nanocaps as its foundation. Sina Kiani et al.^23^, developed a microwave resonant sensor operated at a frequency of 9 GHz and incorporated a band-pass filter within a substrate-integrated waveguide cavity with split ring resonator. In contemporary times, a nascent cohort is emerging, but mainly as a theoretical concept rather than a practical implementation of medical devices, owing to its developing phase of advancement. However, for the sake of this discussion, we will classify these devices as fourth-generation medical devices. This category encompasses noninvasive techniques that provide real-time and continuous monitoring.

**Figure 1:**
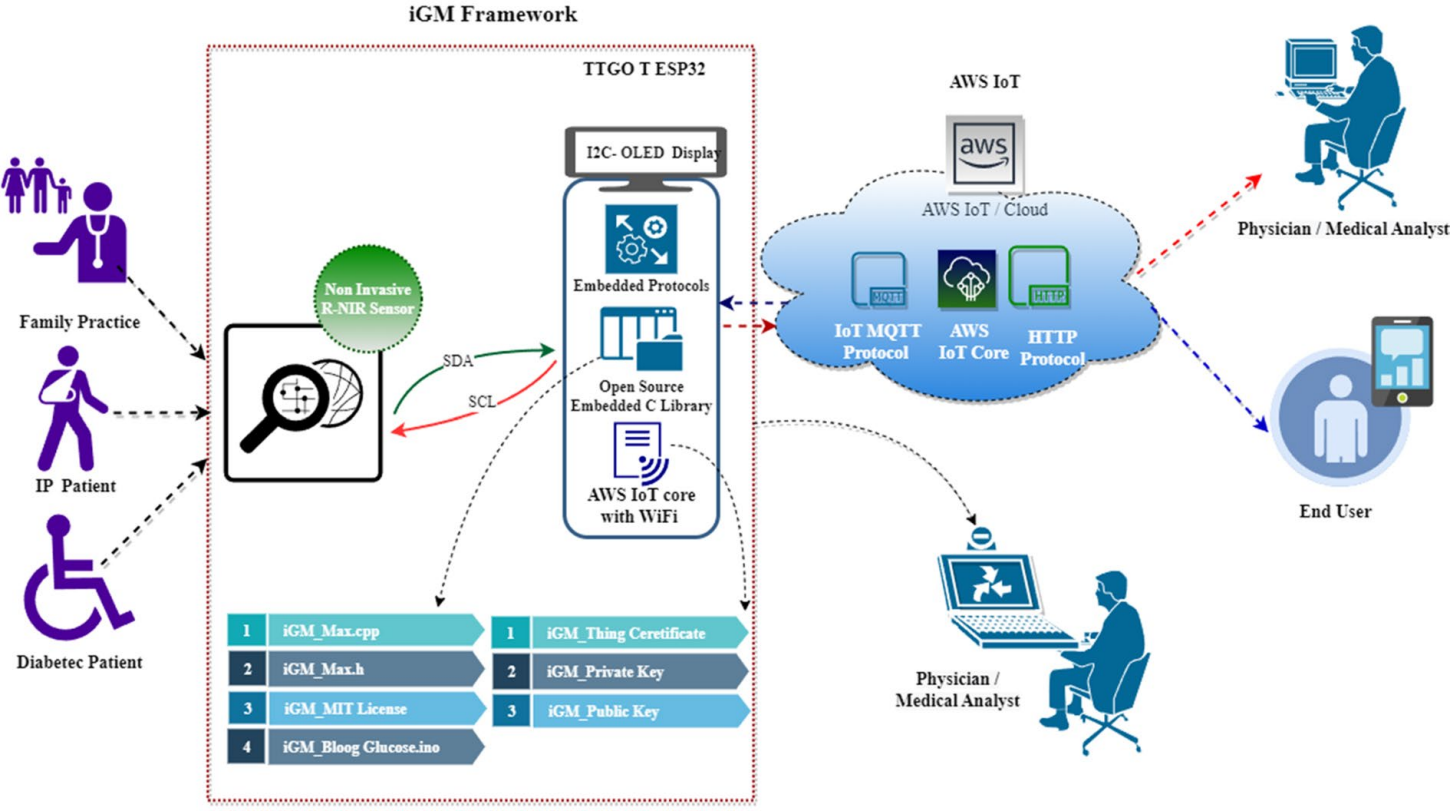
Proposed AWS assisted iGM framework for non-invasive blood glucose monitoring.

Figure 1 depicts the iGM- AWS IoT architecture used in this research. The proposed iGM sensor array has been developed and deployed in ESP32 Module with Organic LED (OLED) display, open-source software with embedded C structure, and Amazon Web Services (AWS) IoT core infrastructure. Recent advancements in sensor technology, wearable devices, and data analytics have enhanced the accuracy of non-invasive blood glucose monitoring. Non-invasive blood glucose monitoring will enhance quality of life, diabetic control, and reduction of infection. Furthermore, it can enhance intellectual capacity of diabetes and improve therapeutic interventions. The developed iGM gadget reduces risk factors for hyper and hypoglycemic conditions. This framework enables patient’s lifestyles and healthcare by effectively using non-invasive sensor devices and open-source software. The important research highlights are discussed below.

1. Designed and deployed an IoT enabled wearable non-invasive real-time blood glucose monitoring using red and near-infrared (R-NIR) spectroscopy.
2. The developed red and near-infrared (R-NIR) sensor framework is integrated into wearable devices for real-time monitoring and data is streamed to Amazon web services (AWS) IoT core architecture.

The rest of this research paper is organized as follows: Sect. “Methodology” investigated the methodology and deployment of the iGM device. Section “Experimental results” presents a detailed analysis and experimental evaluation conducted on the iGM framework. Section “Conclusion” includes the research conclusion and the potential areas for future research in this field.

## Methodology

The authors Abubeker K M^1^, *, Dr. R. Ramani^2^, Sreenivasulu Gogula^3^, Raja Krishnamoorthy^4^, S Baskar^5^, Dr. Sathish Muthu^6^, Dr. Girinivasan Chellamuthu^7^, and Kamalraj Subramaniam^8^ hereby confirm that,

1. We confirm that all methods discussed in this manuscript were carried out in accordance with relevant guidelines and regulations.
2. We confirm that all experimental protocols were done by the non-invasive methods (No finger prick or direct blood samples are not taken for experiment) and hence no approval from institutional and/or licensing committee is required.
3. We confirming that informed consent was obtained from all subjects.

The graphical abstract in Fig. 1 illustrates the open-source-assisted, non-invasive wearable IoT architecture for the iGM framework. The proposed system designed using an ESP32 IoT platform with an inbuilt Organic LED display, open-source software for deploying Red and near-infrared sensors with an integrated C structure, and AWS IoT core. Each component of the iGM gadget is described in-depth in the next section.

### Non-invasive sensor deployment

This research uses red and near IR signals to improve blood glucose monitoring with IoT architecture. Low-Level Light Therapy (LLLT) using R-NIR light is now widely used to treat diabetes problems at the cellular level. According to clinical trials, red light treatment uses 630 nm to 660 nm 810 nm to 850 nm wavelength for NIR signals, it has the broadest range of benefits and no adverse effects. Organic molecules are best detected using near-infrared signals because their bonds absorb wavelengths in the NIR range. Organic chemicals are the main constituents of biological tissue, and NIR can penetrate more profoundly than any other infrared wavelength.

**Figure 2:**
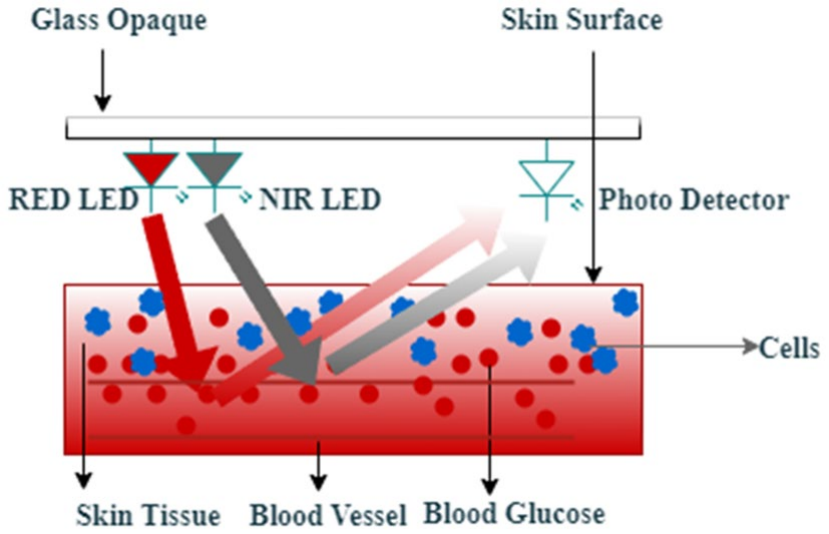
Red and near-infrared (R-NIR) signal absorption characteristics of a human skin.

Figure 2 represents the skin's reflectance characteristic; red signal absorption is high when glucose molecules are present. NIR light can penetrate further into the body than visible light and reach the subcutaneous arteries. It is also possible to do multivariate analysis on the reflected light by collecting it at various optical wavelengths at varying concentrations of blood glucose. To overcome the limitations of earlier approaches, a combined R-NIR biosensor has developed. The suggested system is reliable for continuous practical deployment, and the sensor location on the subject's wrist or earlobe is significant.

Figure 2 illustrates that a decrease in glucose concentration leads to increased scattering, resulting in reduced absorption. Conversely, increased glucose concentration in tissues leads to decreased scattering, therefore increasing tissue absorption. The intensity of reflected light is lower in tissue with higher glucose content due to the increased light absorption by high glucose tissue, as shown by the following equation.

1 $${\text{I}}_{Ri} = {\text{I}}_{Ii} {\text{e}}^{pl} .$$

According to Eq. (1), the reflected light intensity from skin tissues (I_Ri_) equals the product of the reflected light intensity I_*I*i_ and the NIR signal's transit span through the tissue(*l*). Photon absorption in tissue (P_a_) is often calculated as follows:

2 $${\text{P}}_{a} = \, \left( {{2}.{3}0{3}{\mathcal{M}}{\mathbb{C}}} \right),$$

Equation (2) shows that the tissue concentration and molar extension significantly impact how many photons the tissues absorb. Beer-Lambert law states that light intensity through a medium decrease exponentially. Following that, the absorption of NIR spectra is (A_nir_) depicted as follows:

3 $${\text{A}}_{{{\text{nir}}}} = {\text{ log }}[{\text{I}}_{Ii } /{\text{I}}_{Ri} ].$$

The concentration of red (R_bg_) and NIR (NIR_bg_) ranges is determined as:

4 $${\mathcal{C}}_{red} = \, \log \, [R_{bg} /I_{Ii} ],$$

5 $${\mathcal{C}}_{{{\text{nir}}}} = {\text{ log }}[{\text{NIR}}_{{{\text{bg}} }} /{\text{I}}_{Ii} ].$$

Following the measurement of blood glucose, the red (R_ag_) and NIR (NIR_ag_) concentration of is calculated as follows:

6 $${\mathcal{C}}_{{{\text{red}}}} = {\text{ log }}[{\text{R}}_{{{\text{ag}}}} /{\text{I}}_{Ii} ],$$

7 $${\mathcal{C}}_{{{\text{nir}}}} = {\text{ log }}[{\text{NIR}}_{{{\text{ag}} }} /{\text{I}}_{Ii} ].$$

The spectral concentrations in the red and NIR regions shift as seen in the following Eqs. (8) and (9).

8 $$\Delta_{{{\text{red}}}} = {\text{ log }}[{\text{R}}_{{{\text{ag}}}} /{\text{R}}_{{{\text{bg}}}} ],$$

9 $$\Delta_{nir} = \, \log \, [NIR_{{{\text{ag}}}} /NIR_{{{\text{bg}}}} ],$$

To minimize calibration time, a full-day blood glucose concentration change estimate was evaluated. LED and photodiode are placed next to one other on the same tissue site for a diffused reflectance measurement.

**Figure 3:**
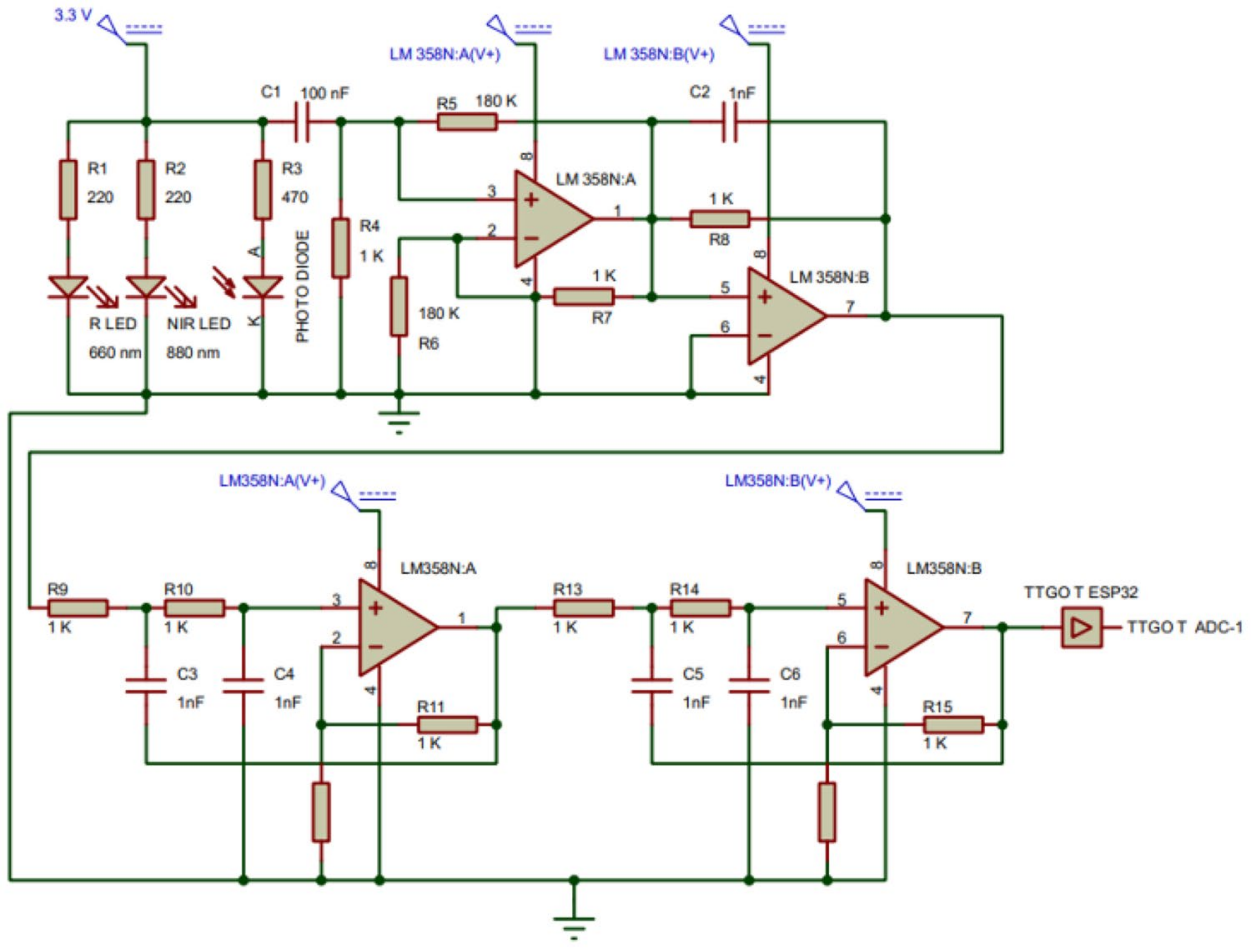
Circuit schematic of the developed iGM gadget.

A sensor has placed on the wrist skin with a 3 mm spacing with device body for better accuracy and reflection without affecting the external noises and disturbances. Interconnected systems of superficial veins make up the wrist. Effective blood sampling is guaranteed by positioning the sensor in this area. By keeping the sensor in constant touch with the skin, the wrist reduces the impact of movement artefacts on the sensor's accuracy. In Fig. 3, a sensor array includes a NIR LED (880 nm), a trans-impedance amplifier and a photodiode (800–1700 nm). Absorption and dispersion reduce light's intensity when it comes into contact with living tissue. The wrist reflects an attenuated and distorted light that is picked up by the receiver. Light noise is reduced by noise filtering components, and then the received signal is amplified so that it can travel farther despite its low strength. ESP32's 12-bit analogue to digital converter (ADC) converts the desired signal into an electric current, and a trans-impedance amplifier (TIA) using the Op-Amp converts the photodiode current to voltage. TIAs have a significant propensity to oscillate at high frequencies over the gain-bandwidth product because of their high gain operation. A 0.8Hz High Pass Filter (HPF) and a 10Hz Low Pass Filter (LPF) are used to eliminate the DC component from the resulting R-NIR signal and to reduce the power line and other signal disturbances that might interact with the PPG and give us inaccurate data, respectively. The iGM framework provided an IoT-based system by merging the AWS IoT core with a personalized sensor circuit despite the rarity of the methodologies for IoT-based systems.

Various non-invasive blood glucose measuring devices are compared in Table 1. There are a wide variety of photoplethysmographs in use, some of which contain artificial intelligence and machine learning algorithms to predict blood glucose. Reflection and absorption spectroscopy with fingertip measurements is the most flexible technique to apply NIR spectroscopy. Researchers have yet to develop an accurate and flexible device, and the vast majority of them are still working on PPG-based systems. The IGM framework provided an IoT-based system by merging the AWS IoT core with a bespoke sensor circuit despite the rarity of the methodologies for IoT based systems. Compared to other devices on the market, it boasts a high degree of precision and a low rate of inaccuracy.

**Table 1:** Performance evaluation of different technologies used in non-invasive blood glucose measurement.

SI. no.	Methodology	Mode of operation	Type of gadget	Type of architecture
1	Proposed iGM gadget using IoT	Real time IoT based	Wearable and wrist based.	AWS IoT Core
2	Noninvasive blood glucose monitor via multi-sensor fusion^24^.	Automatic	Wearable and fingertip based	Hybrid and multi-layer embedded sensors
3	Blood glucose and blood pressure from a PPG by means of machine learning techniques^25^.	Artificial intelligence based	Non wearable and fingertip based	A multilayer CNN, SVM and random forests.
4	PPG based machine learning approach^26^.	Automatic	Wearable and fingertip based	Embedded module and custom sensors
5	Near-infrared spectroscopy based on PLSR combines SAE deep neural network approach^27^	Manual	Non wearable and fingertip based	Hybrid model of PLSR and SAE with DNN
6	Fingertip capillary dynamic near infrared spectrum (DNIRS) measurement combined with multivariate linear modification algorithm ^28^.	Automatic	Portable and fingertip based	Dynamic NIR spectrum method
7	Wavelength-modulated differential photo thermal radiometry^29^.	Automatic	Non wearable	Embedded sensors
8	Visible-NIR optical biosensor^30^.	Automatic	Wearable band	Embedded sensors

### Open source library development

The future of open source development will be driven by the need for the Internet of Healthcare Things (IoHT). There has been a dramatic increase in the use of Tactile Internet (TI) technology in recent years to speed up real-time data delivery from many applications, including healthcare.

**Figure 4:**
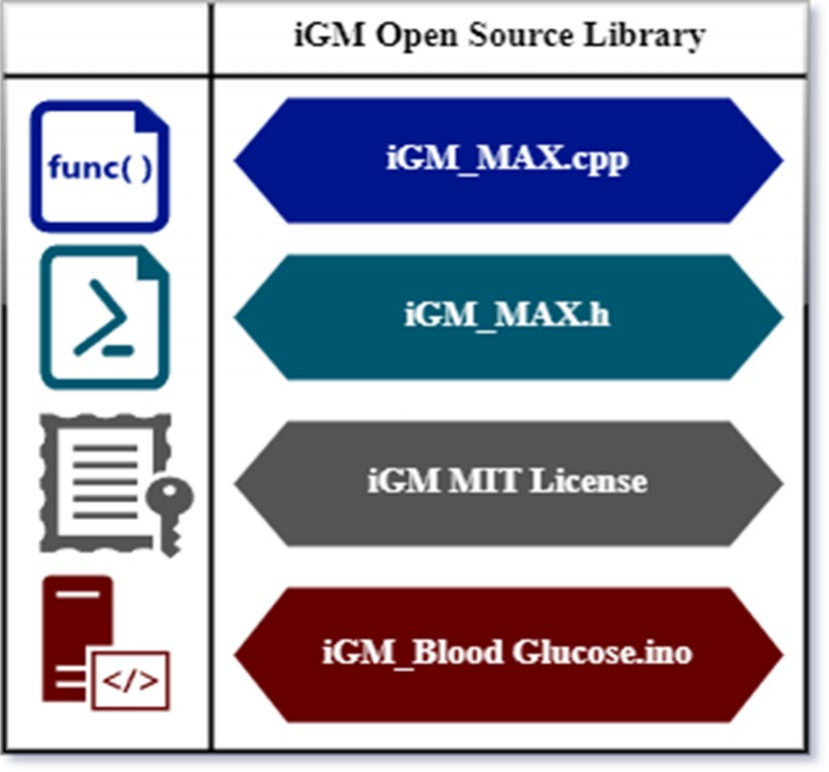
Structure of iGM open source library licensed by MIT.

Figure 4 illustrates the open-source structure of the proposed iGM framework developed with embedded C, licensed under Massachusetts Institute of Technology (MIT). Additional functionality has been added to the sketch or source code using C++ libraries. iGM_Max.cpp has the programming library, iGM_Max.h comprises the headers and one text file that lists all of the keywords used in library development. Custom functions in the iGM_Max.cpp files include RValue () and NIRValue () functions, which are used to return the red and near-infrared reflection values from the inner wrist and earlobes of the patient.

### AWS IoT core

A message queuing and telemetry transport (MQTT) is a machine-to-machine (M2M) communication infrastructure and a Secure hypertext transfer protocol (HTTP) are two IoT services of AWS IoT Core for publishing and subscribing to messages. Smart wearable sensors may send and receive data with limited bandwidth, making the ability to exchange IoT data effectively. The iGM framework has integrated with a TTGO ESP32 IoT development board powered by an Xtensa dual-core 32-bit microprocessor with a 240 MHz operating frequency, with organic LED display. Wi-Fi and Bluetooth are incorporated into the ESP32, making it a suitable Iot platform for server less connections to AWS IoT Core devices. AWS offers various services, including the MQTT library, and AWS IoT core communication establishment. The first step is to construct the AWS IoT Things, IoT things should record physical actions; IoT Policy is a JSON document specifying authorized operations. AWS IoT core receives sensor data through the MQTT protocol, and the open-source library analyses it.

**Figure:**
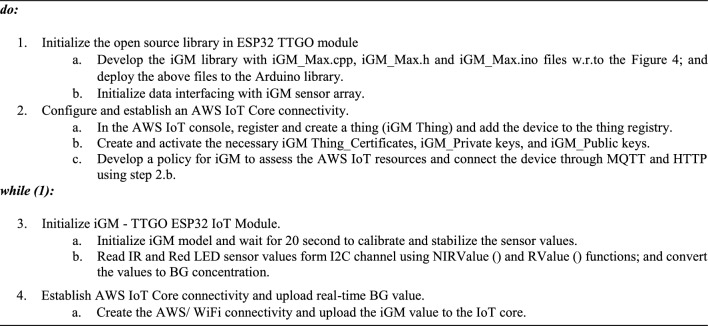
Algorithm 1.

ESP32 has traditionally been the best choice for developing IoT based wearable applications because of its flexibility in open source programming, IoT connectivity, and low-power design with an OLED display. The manuscript investigates the deployment of an open-source library for iGM based on the Arduino IDE and includes a custom module that has been specifically defined for the derived R-NIR sensors.

## Experimental results

The blood glucose level is a crucial indicator of diabetes control and is expressed as milligrams per deciliter (mg/dL). Diabetic patients can maintain a fasting blood glucose level of 80 to130 mg/dL and a postprandial blood glucose level of less than 180 mg/dL. A blood test called glycosylated hemoglobin (HbA1c) can estimate how stable a person's blood glucose has over the previous two to three months. Patients with diabetes should aim for an HbA1c level of 48 mmol/mol or less (6.5%). The normal range for HbA1c in people with type 2 diabetes is between 5.7 and 6.4 %. This research outcome shows the average blood glucose (ABG) levels in people with diabetes have strongly correlate with their HbA1c values. Several variables determine the HbA1c level of an individual such as, age, general health, pregnancy status, and diabetes background.

Table 2 shows the results of seven tests used to evaluate the effectiveness of the proposed hardware. The subjects are selected with equal priority for men and women with different age groups including non-diabetic, prediabetes and diabetic patients. During evaluation phase, the efficacy of the iGM device has tested within a day with a one-hour interval, and researchers consider the various levels of participants by considering age, diabetes history, HbA1c, etc. In contrast to CGM, the proposed iGM makes use of near-infrared technology to detect blood glucose without intrusive procedures. It can be employed in the earlobes, finger tips, wrist, and even soft tissues of the body, without contact with the patient's body. The observed glucose level is shown on the wearable device's OLED screen, and it can additionally be broadcast to AWS IoT Core cloud enabling remote monitoring. Table 2 contrasts the test results of 7 individuals picked at random from various groups, such as those pre-diabetic, non-diabetic, and type 1 or type 2 diabetes. During a 10-hour fast, the iGM device's accuracy is 98.82 %; two hours after breakfast, it's 98.04 %. Test participants are restricted to 15, and the error rate reach up to 1.18 % and 1.96 %, respectively, when compared to current best practices. Equation (10) presented the formula for calculating the error percentage (Er).

10 $${\text{Er}} = \, [(IGM_{BG} - {\text{Re}} f_{RG} ) \, / {\text{Re}} fBG] \times {1}00.$$

**Table 2:** Estimated blood glucose levels throughout the day using iGM with pre-diabetic subject (S1,S6), non-diabetic subject (S2) and diabetic subject (S3,S4,S5,S7).

Subjects	Age & gender	Diabetic history (in year)	Diabetic status	BG level –mg/dl				HbA1c
				Fasting (8 AM)	2 Hr. after breakfast	Pre-prandial (before meals 1 PM)	Post prandial (1.5 Hr. after meals 2.30 PM)	
S1	39-M	2	Prediabetes	128	164	134	172	5.8
S2	41-F	0	Nil	110	127	119	143	5.2
S3	53-M	14	Diabetic	169	231	200	256	6.9
S4	65-F	4.5	Diabetic	221	248	234	256	11.8
S5	48-F	6	Diabetic	183	221	205	244	7.3
S6	43-M	1.5	Prediabetes	115	153	129	168	5.4
S7	51-F	7	Diabetic	193	230	189	245	7.6

Here, *iGM*_*BG*_ and *Ref*_*BG*_ are the BG value from iGM and Accu-Check Active respectively.

Accuracy (Ac) of the IGM device is determined by first determining the error rate (Er) using Eq. (10). The percentage error rate is calculated by dividing the difference between the iGM and blood glucose values by the Laboratory testing, as shown in Eq. (11).

11 $${\text{Ac}} = { 1}00\% \, - {\text{Er}}.$$

By comparing the readings with those from an Accu-Check Active blood glucose monitor, the researchers were able to confirm that the proposed device have superior clinical accuracy. A total of three different groups of subjects participated in the tests; healthy volunteers between the ages of 30 and 40 without a history of blood glucose; diabetes patients between the ages of 35 and 55, including men and women. Accu- Check Active takes invasive blood samples from the subject's finger while iGM collects real-time blood glucose data from patient’s wrist and earlobe. Blood glucose levels is measured in the morning before breakfast, lunch, and dinner, and two hours after each meal and snack. The real time BG values are monitored on IoT enabled wrist watch with OLED display as well as on the AWS cloud platform. The ESP32 microcontroller is a powerful platform for developing wearable applications due to its open-source development environment, IoT connectivity, and low-power design with OLED display. The manuscript investigates the deployment of an open-source library for iGM based on the Arduino IDE and includes a personalized module that has specifically defined for the developed R- NIR sensors.

This research used different testing with various subject matter and compare with current techniques to empirically support the claim that the suggested approach enhances glucose monitoring accuracy using non-invasive sensors. Independent validation, statistical significance of the iGM device, reference measurements, etc., have all been performed in this research.

Table 3 compares the performance of the developed iGM hardware with the prominent research in this area. When evaluating CGM devices, it is essential to consider individual preferences and needs, personal lifestyle, daily activities, and specific medical requirements. The CGM device's readings align with reference blood glucose measurements from traditional finger stick tests or lab-based methods. It is observed that the iGM device does not require frequent calibration. Researchers have compared the accuracy of the suggested approach to that of many other laboratory tests and non-invasive blood glucose measurement techniques. Compare the upfront and ongoing costs of the iGM system; the iGM market is continually evolving, be sure to refer to the most current and authoritative sources for the latest information on device performance comparisons. By comparing the readings with those from an Accu-Check Active blood glucose monitor, the researchers were able to confirm that the suggested device had superior clinical accuracy.

**Table 3:** Performance comparison of the iGM and other CGM devices available in the global market.

	Mechanism	Targets	Advantages	Disadvantages	Costs	Versatility
iGM	Non-invasive NIR based.	Type 1 & 2 diabetes.	IoT based monitoring, battery life up to 6 months.	Real time data can be updated only with internet facility.	30 $	Wearable and wrist or earlobe based BG monitoring.
CGM- abbott diabetes care Ltd, UK^31^	Invasive-interstitial type.	Type 1 & 2 diabetes.	Measure interstitial glucose and stores 90 days of glucose data.	Separate sensor and reader. Expensive and 10-15 days’ battery life.	306 $	Attached to the body with separate display with WiFi connectivity.
One care meta sense^32^	Invasive-interstitial type.	Type 1 & 2 diabetes.	Self-activate the sensor.	Sensor backup for up to 14 days.	66 $	One touch activation and health coach through app
Ultrahuman M1^33^	Invasive-interstitial type.	Type 1 & 2 diabetes.	Connects to aerobic activity.	Relaying glucose data to the app requires NFC connection.	303 $	Glucose-based meal score and optimization tips.

Blood glucose levels in the iGM before one hour of breakfast to two hours after supper (08 am to 10 pm) for diabetes, healthy individuals, are shown in Table 4. The developed iGM blood glucose monitor has evaluated using one-hour intervals for analyzing the accuracy and performance of the monitor. One-hour intervals may effectively observe fluctuations in blood glucose levels, ensuring a temporal resolution, which may help identify patterns and fluctuations in glucose compared with less frequent monitoring. For diabetic patients, blood glucose levels can have fast fluctuations, mostly after meals. This allows ability to detect postprandial glucose spikes and determine the delay between fluctuations in blood glucose levels and the monitor's recorded values. This approach is suitable for statistical analysis, facilitating the evaluation of the monitor's accuracy and precision. To obtain real-time readings, the Accu-Check Active (ACC) device and laboratory testing are used in this research. This comparison evaluated the accuracy and reliability of blood glucose measurements obtained from the iGM system, ACC device, and laboratory tests (LAB) results.

**Table 4:** Measured blood glucose levels using iGM, Accu-Check Active (ACC) device and laboratory tests (LAB) results for subjects S1 to S3.

Time → (24 Hr.)	8	9	10	11	12	13	14	15	16	17	18	19	20	21	22
S1-(iGM)	128	142	164	158	139	134	159	172	143	145	132	130	141	130	130
S1-ACC	129	141	164	157	140	133	159	170	141	144	133	130	142	129	130
S1-LAB	128	140	165	158	1490	133	159	171	142	145	132	132	140	131	130
S2-(iGM)	110	121	127	129	126	119	131	143	120	129	120	119	129	122	119
S2-ACC	110	120	125	127	125	120	130	144	119	130	121	120	130	`120	120
S2-LAB	111	121	126	129	125	120	131	142	120	128	121	120	129	123	121
S3-(iGM)	169	201	231	226	209	200	239	256	221	232	218	214	232	211	204
S3-ACC	170	200	230	227	210	200	240	258	225	230	220	217	233	214	203
S3-LAB	170	200	230	225	210	200	240	255	220	233	220	214	232	210	204

### Accuracy and precision

- iGM: The iGM system provides continuous glucose monitoring, eliminating the need for frequent finger pricking. It measures interstitial fluid glucose levels and has shown promising accuracy compared to laboratory tests. From Table 3, the proposed system has high accuracy and reliability in BG measurement.
- Accu-Check: The ACC glucometer measures capillary blood glucose levels and is widely used for self-monitoring by individuals with diabetes. It was extensively validated and proven accurate and reliable in many studies. Still, as per the results, it needs more accuracy in chemical test results in the laboratory.
- LAB: Laboratory tests involving venous blood samples are considered the gold standard for blood glucose measurement. They offer high accuracy and precision, but the process involves more time and resources than the other two methods.

### Convenience and ease of use

- iGM: The portable and self-wearable nature of the iGM system offers unparalleled convenience, as it continuously monitors glucose levels without the need for frequent manual testing. However, its initial placement does not require a medical procedure and professional calibration.
- ACC Device: The ACC glucometer is portable, user-friendly, and requires a small amount of blood for testing. It is ideal for daily self-monitoring and provides instant results.
- LAB: Laboratory tests require individuals to visit a healthcare facility for blood sample collection, which may not be as convenient as using the other two methods for daily monitoring.

### Device cost

- The iGM system's initial cost, including the deployment procedure and device, can be relatively less than traditional glucometers. From Table 2 we can see that the cost of the product is only ten percentage of the devices available in the market.
- ACC Device: The ACC glucometer is less affordable in low-income countries regarding initial cost and ongoing expenses for test strips.
- LAB: Laboratory tests are typically covered by health insurance, but they can incur higher costs when performed frequently.

### Continuous monitoring

- The iGM system monitors glucose levels and provides a continuous glucose profile, offering valuable insights into glucose trends over time.
- ACC Device: The ACC glucometer provides instant blood glucose readings, but it does not offer continuous monitoring.
- LAB Results: Laboratory tests provide a single snapshot of blood glucose levels at the time of the test, limiting their ability to capture fluctuations throughout the day.

The blood glucose monitoring method choice depends on individual preferences, needs, and the level of diabetes management required. The iGM system offers the advantage of continuous monitoring but comes with lower initial costs and no processing cost required. Ultimately, consultation with healthcare professionals can help individuals select the most suitable method for their diabetes management plan.

**Figure 5:**
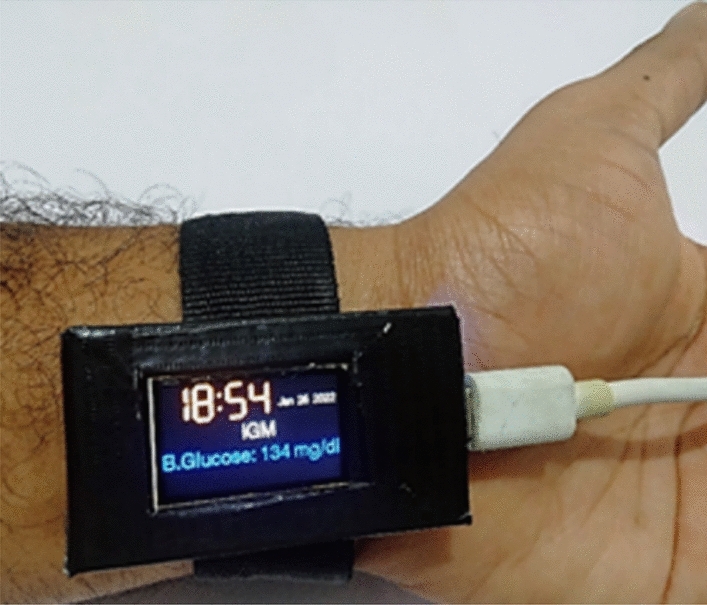
An IoT-enabled real-time blood glucose monitoring system (iGM) deployed in diabetic patient using ESP32 IoT module with organic LED display.

Figure 5 shows an iGM device developed with R-NIR spectroscopy is deployed in patient’s wrist with OLED display. A small data-collection window of 20 seconds was chosen to guarantee a steady but continuous system further. Inter-integrated circuit (I2C) is an in-built protocol used for short-distance communication in embedded system components, and it is used to connect the iGM model's NIR and RLED reflection brightness to the ESP32 Iot platform. Some earlier techniques developed to gather signals from a person's fingers or arms have proven helpful in blood glucose measurement systems. Fingers can be used for invasive and non-invasive blood glucose measures; in the former scenario, a drop of blood has been extracted from a capillary. On the other hand, several of the alternatives that have been presented are invasive and limit the subject's mobility. So that the proposed sensor system can function as an IoHT-compliant wearable device using non-invasive NIR technology.

**Figure 6:**
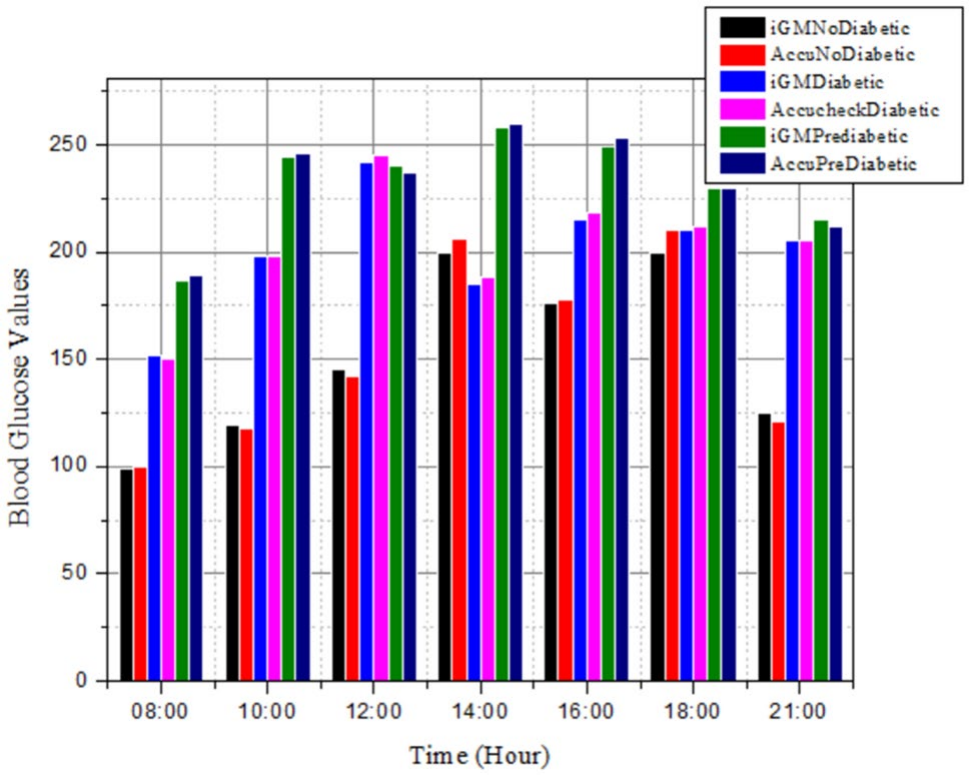
iGM vs Accu Check Active blood glucose test results comparison for non- diabetic, pre-diabetic and diabetic patients. The iGM glucose measurement on the wrist and Accu check active on patients’ fingertip.

Figure 6 depicts the performance comparison between the iGM and Accu Check Active blood glucose monitor. The developed iGM would function as portable device for constantly measuring blood glucose levels of ambulatory, ICU, and pregnant women without calibration. This Wearable, non-invasive glucose monitors have improved diabetic management and enhance the lives of millions of diabetic patients worldwide. The iGM can be used to make prompt modifications to insulin dosages and lifestyle choices with artificial intelligence and machine learning algorithms. In addition to this better diabetes management is possible to analyze individual glucose patterns and forecast future glucose trends. The use of non-invasive glucose monitoring equipment can be extended to health and fitness apps to track the changes in food and exercise affect in blood sugar levels.

## Conclusion

The proposed iGM framework helps for diabetic patients to keep continuous monitoring blood glucose levels for improved treatment and enhanced life styles. The measured blood glucose levels can be monitored remotely and treated promptly using information sent to physicians via IoT devices. The wearable iGM gadget uses a customized sensor with Red Near InfraRed (R-NIR) spectroscopy and an I2C protocol interface. Open-source frameworks improve the accuracy, user adaptability and creativity among healthcare professionals, facilitating the framework's enhancement and modification. The research findings of the iGM blood glucose monitor and the invasive state-of–art methods are compared with three groups of subjects throughout the research by considering the factors like age, diabetes status, and HbA1c. The signal quality and streaming capabilities of the iGM gadget best suited for ICU patients, and isolated patients. The main challenge of this product is the battery backup, it can take up to 23 days, and may improve by using Li-ion batteries. Future developments will integrate Nano sensors and deploying artificial intelligence and machine learning algorithms will enhance the gadget performance. Also data visualization tools will heighten the user experience in interpreting glucose data and facilitate proactive decision-making.

## Data Availability

The datasets used and/or analysed during the current study available from the corresponding author on reasonable request.
